# Ureteroscopy during pregnancy

**DOI:** 10.4103/0970-1591.56173

**Published:** 2009

**Authors:** Michelle J. Semins, Brian R. Matlaga

**Affiliations:** James Buchanan Brady Urological Institute, The Johns Hopkins University School of Medicine, Baltimore, Maryland, USA

**Keywords:** Calculi, kidney, pregnancy, ureteroscopy

## Abstract

Urolithiasis during pregnancy is an uncommon, but a serious medical problem. Options for the treatment of pregnant women with obstructing stones include ureteral stent placement, percutaneous nephrostomy tube placement, and ureteroscopic stone removal (URS). Although ureteral stent and nephrostomy tube placement have been the historically standard treatment option for pregnant women with obstructing stones, there is an emerging collection of literature that reviews the safety of URS for pregnant women. We performed a systematic review of MEDLINE and EMBASE from January 1966 through April 2009 to identify all literature on URS in pregnant women. Herein, we review the literature on URS during pregnancy, with a focus on the safety of this approach. We conclude that URS is an appropriate intervention in the pregnant population with urolithiasis; in all cases the procedure should be performed on a properly selected patient by a surgeon with appropriate experience and equipment. With such an approach, complication rates are low and success rates are high. A multidisciplinary approach should be emphasized as a key to a successful outcome.

## SCOPE OF THE PROBLEM

Although urolithiasis during pregnancy is an uncommon problem, it can nonetheless pose many risks to both the pregnant mother and the unborn fetus. Over the past several decades, there has been a significant increase in the prevalence of nephrolithiasis among the general population; the lifetime risk for this disease is now reported to be between 10 and 15% in the United States.[1] Given the rapidly increasing prevalence rate, it is unlikely that the pregnant population will be spared from this trend. At present, symptomatic urolithiasis has been reported to affect between 1 in 200 to 1 in 1500 pregnant women.[2–6] The latter number is likely an underestimate of overall stone incidence in pregnancy as many pregnant women may harbor undiagnosed stones. Urolithiasis in pregnancy may be more common in multiparous women, and more commonly present during the second and third trimesters. Both the right and left kidneys have been reported to be affected equally.[7,8]

The specific risks associated with renal colic during pregnancy are well characterized. Much of this literature, though, has been restricted to publications targeted primarily toward obstetricians, rather than urologists. Two small series were among the earliest descriptions of the effects of urolithiasis on pregnancy. Drago and associates reported on 9 pregnant women admitted for symptomatic kidney stones and 6 of them experienced preterm labor.[9] Hendricks and associates similarly reported on 15 pregnant women admitted for nephrolithiasis and 6 of them ultimately had preterm delivery.[10] Several analyses of large databases confirmed the findings reported in these small series. Specifically, Swartz and associates analyzed the state of Washington's hospital discharge data from 1987 through 2003.[11] They found that women admitted for nephrolithiasis had a significantly greater (adjusted odds ratio 1.8) risk of preterm delivery compared to women without stones. In 2003, Lewis and associates also reviewed over 21,000 deliveries in their database, and found that for the 86 patients diagnosed with a stone during pregnancy, there was an increased risk of preterm premature rupture of membranes (2.9% in non-stone patients vs. 7% in stone patients).[12] Preterm premature rupture of membranes is associated with an increased risk of morbidity and mortality to the newborn. In contradiction to the aforementioned reports, a group from Hungary studied a large population-based dataset from 1980 through 1996 and found no higher risk for adverse birth outcomes, specifically congenital anomalies, preterm birth, and low birth weight in newborns of pregnant women with nephrolithiasis.[13] Although this report is reassuring, it must be recognized that the source data for this study were obtained from patient questionnaires, and there were different compliance rates between the study and control groups. Taking these reports all together, though, nephrolithiasis during pregnancy remains a real concern. The subject of stone disease during pregnancy is particularly timely in the modern era, given the increasing incidence of kidney stone disease.

The pregnant patient with nephrolithiasis can be a diagnostic challenge, as there is a need to minimize radiation exposure for this unique group. Additionally, the presenting symptoms of renal colic can be atypical in a pregnant patient, leading to further confusion. In fact, up to 28% of pregnant patients with renal colic and urinary calculi are misdiagnosed with medical conditions like appendicitis or hydronephrosis of pregnancy.[2] Because the pregnant patient may be at increased risk for adverse events from nephrolithiasis than the general population, and the clinical situation is complex, clearly defining the management algorithm of these patients is important. Specific challenges inherent in caring for the pregnant patient include a need to avoid radiation exposure, as well as general anesthesia; furthermore, medication choices can be limited for these patients. Herein, we discuss the management of urolithiasis during pregnancy with a focus on ureteroscopy (URS), based on a systematic review of MEDLINE and EMBASE from January 1966 through April 2009 identifying all literature on URS in pregnant women and stones in pregnancy. We first review URS in pregnancy in depth, and then shift focus to discuss alternative management strategies in pregnant patients with symptomatic urolithiasis.

## MANAGEMENT

Most pregnant women with symptomatic kidney stones can be managed conservatively during pregnancy. In fact, between 40 and 80% of patients will spontaneously pass their stone without complication.[14] However, there are certain pregnant women with a kidney stone, though, who may suffer from infection, persistent pain, progressive renal obstruction (unilateral or bilateral), obstruction of a solitary kidney, or obstetric complications (i.e. premature labor or pre-eclampsia). Such clinical scenarios will necessitate a more definitive approach than expectant management. Such a strategy will be targeted at either a temporizing procedure to ensure drainage of the kidney, or a definitive procedure with removal of the offending stone.[6]

Historically, ureteral stents and percutaneous nephrostomy drains were the only acceptable options for pregnant women requiring an intervention for an obstructing stone.[15] With the rise of shock wave lithotripsy, case reports have appeared describing the application of this technique to pregnant women. However, it should be noted that pregnancy remains a contraindication to this approach, as it increases the risk of placental displacement, miscarriage, and congenital malformation.[16] Both stents and nephrostomy drains will effectively decompress an obstructed collecting system. However, both methods do have inherent disadvantages, including encrustation of the prosthetic device, as well as the temporary nature of the intervention necessitating a definitive procedure in the future. As clinical experience with URS has increased, there has been increased interest in applying this definitive treatment approach to the pregnant population. However, it should be noted that the literature detailing URS during pregnancy is limited, and entirely composed of single institution case series.[17–32]

**Table 1:** Complications reported in published literature

References	n	Complications
Juan[28]	3	None
Akpinar[29]	7	2 – post-operative pain
Khoo[21]	5	None
Yang[31]	3	None
Lifshitz[27]	6	None
Watterson[19]	8	None
Lemos[20]	14	None
Shokeir[26]	10	2 – urinary tract infection
Parulkar[18]	4	None
Scarpa[23]	15	None
Carringer[24]	4	None
Ulvik[22]	25	1 – ureteral perforation
		1 – premature uterine contractions
		3 – urinary tract infection
Vest[30]	2	None
Rittenberg[25]	2	None
Travassos[17]	9	None
Rana[32]	19	None

## URETEROSCOPY

In recent years, there have been increasing numbers of reports detailing URS for pregnant women. To some extent, recent improvements in surgical technology are responsible for the increased utilization of URS in the treatment of pregnant women. Among the endoscopic advances, perhaps the most important is miniaturization of the endoscope itself. As recently as one decade ago, the standard ureteroscope was of an 11 French diameter. The diameter of modern ureteroscopes has been reduced dramatically, such that standard available ureteroscopes are of 6–8 French diameter. Consequently, ureteral dilation is rarely necessary when using a modern ureteroscope, and accessing the renal collecting system is now a more straightforward, safe, and expedient process than it was with the previous generation of endoscopes. In addition to the miniaturization of the endoscope itself, graspers and other implements used to manipulate the stone have been similarly reduced in size. These miniaturized devices permit the atraumatic manipulation of stones within the upper urinary tract. Any endoscopic implement passed through the working channel of an ureteroscope will have a deleterious effect on the function of the ureteroscope. In particular, ureteroscope deflection and irrigation flow will be adversely affected. However, the smaller the diameter of the device, the less of an adverse effect it will have; thus illustrating the importance of device miniaturization.

Overall, complications in pregnant women undergoing URS are uncommon. However, it is important to know whether being pregnant affects a patent's risk for adverse events associated with URS. As there has never been a randomized controlled trial comparing URS in pregnancy to ureteral stent or percutaneous nephrostomy placement, our knowledge of the risk of this practice is gained from an analysis of retrospective case series. A review of the published literature on the experience with URS of pregnant women finds a total of 16 published case series and one meta-analysis of 14 reports completed prior to the meta-analysis[4,17–32] [Table 1]. The aim of the meta-analysis was to define the safety of URS during pregnancy, by comparing complication rates of pregnant women undergoing URS to complication rates of the non-pregnant undergoing URS. Specifically, the existing literature on URS in pregnant women was analyzed, and the complication rates quantified. These rates were then compared to the American Urological Association and European Urology Association's Guidelines on the Management of Ureteral Calculi. Complications were stratified by Clavien criteria, which is a validated classification system that allows a standardized and reproducible means of comparing the complications among the source studies.[33] The 14 reports were comprised of 108 women. A total of 9 complications were encountered, which were generally considered to be minor and classified as Clavien 1 or 2. There was one patient in the meta-analysis, though, who required temporary ureteral stent placement. When these data were compared to the data reported in the AUA/EAU Guidelines document, there were no significant differences in the complication rates. Among both the pregnant and the non-pregnant cohorts, complications were uncommon, and it appeared that the pregnancy status of the patient did not affect the complication rate. An important caveat about this meta-analysis is recognition of the selection bias that may be inherent in these studies.

A more recent study by Travassos and associates was not included in the aforementioned meta-analysis.[17] These authors reviewed their experience with 19 pregnant women suffering from obstructing ureteral calculi. The women were initially treated with a conservative approach, with analgesia, hydration, and antibiotics. In 9 patients, this approach failed, and URS was performed for either persistent pain or hydronephrosis. In all cases a semi-rigid ureteroscope was used, and the authors describe a 100% stone free rate, with no obstetric or surgical complications. Additionally, to mitigate concerns of exposure to ionizing radiation, no fluoroscopy was used for these cases.

Rana and associates also reported on a 10 year experience with URS during pregnancy.[32] They retrospectively analyzed 19 pregnant women with obstructing ureteral calculi. Patients underwent semi-rigid URS, with pneumatic lithotripsy used when necessary. All patients tolerated the procedure well with no obstetric or surgical complications and 79% stone fragmentation rate reported. Of note, the patients did undergo general anesthesia. A total of five patients required balloon dilation of the ureteral orifice to obtain access. Also, there were two patients who developed a retained, encrusted stent although it is unclear how long the stents were in place prior to this complication.

A recent literature review by Srirangam and colleagues concluded that URS is the “procedure of choice” for pregnant women with obstructing ureteral stones, assuming appropriate endourologic skills and adequate equipment are available.[34] Infection and sepsis is of course a contraindication to URS. Biyani and Joyce outlined other contraindications to URS and also concluded stones greater than 1cm, multiple calculi, sepsis, a transplant kidney, poor equipment and a solitary kidney may be contraindications to immediate URS during pregnancy.[15] Table 2 outlines what we consider absolute contraindications to URS during pregnancy.

**Table 2:** Absolute contraindications to ureteroscopy during pregnancy

Sepsis
Infection
Inadequate equipment
Inexperienced surgeon
Large stone burden

## TREATMENT ALTERNATIVES

The alternatives to URS are ureteral stent or nephrostomy tube placement. As previously noted, SWL should not be performed on a pregnant woman. Both nephrostomy drains and ureteral stents will drain an obstructed collecting system. However, both of these interventions require maintenance of the stent and tube throughout the duration of pregnancy and necessitate a definitive treatment procedure in the post-partum period. An often noted advantage of nephrostomy drainage or ureteral stent placement is that these procedures may be performed without general anesthesia. However, in recent years, many of the reports of URS in pregnant women have also utilized local or regional anesthesia.[20,22,24–26,28]

### Nephrostomy tube placement

Percutaneous nephrostomy tubes are considered to be a reasonable treatment option for pregnant women with obstructing renal calculi, as they will effectively drain an obstructed collecting system. However, when nephrostomy tubes are used in the pregnant population, there are some limitations that should be noted. In 1992, Kavoussi and associates reported that the majority of pregnant patients managed with nephrostomy tubes will require tube exchange due to occlusion from debris.[35] In their series, the authors noted that one-third of the patients ultimately required removal of the nephrostomy tube due to recurrent tube obstruction, fever, or pain. In 2004, Khoo and associates also reported on a series of 29 pregnant women managed with nephrostomy drainage.[21] Over half of these patients required unplanned tube exchanges, replacements, or flushings, due to either dislodgement or obstruction. In addition to the medical difficulties noted in the above series, nephrostomy drains may also have an adverse effect on patients' quality of life, as they often are associated with painful symptoms.

### Ureteral stent placement

While some advocate decompression of the collecting system with a nephrostomy tube placement, others report that ureteral stents are the optimal treatment for pregnant women with an obstructing stone. Like nephrostomy tubes, ureteral stents do effectively drain an obstructed collecting system. However, they, too, have limitations. Accelerated encrustation of ureteral stents occurs in this population, likely secondary to the changes in urinary chemistry that occur during pregnancy, such as hypercalciuria and hyperuricosuria.[5,15] Ureteral stents placed in pregnant women must generally be exchanged every four to six weeks to avoid encrustation and potential obstruction, necessitating the repeated cost and morbidity of repeated procedures. Indwelling ureteral stents themselves are associated with an increased risk for bacteriuria, urinary tract infection, as well as stent migration, all of which may have an adverse effect on the pregnancy.[35,36] The multiple procedures, morbidities, and pain associated with ureteral stents can all have a negative impact on a patient's quality of life. For these reasons, URS may be the preferred treatment in the properly selected pregnant patient, particularly as the device technology and surgical technique continues to improve.

## CONCLUSIONS

URS is a safe and effective treatment option for the pregnant woman with an obstructing stone who has failed conservative treatment options. The complications associated with URS in the pregnant population are uncommon, and when they do occur they are reported to be, in general, minor. Alternative therapies for the pregnant woman with an obstructing stone, such as nephrostomy drainage or ureteral stent placement, are associated with an increased risk for infection, obstruction, drain dislodgement, and are not definitive therapies. Although URS should be considered as an appropriate treatment option for such pregnant women, several caveats should be noted. In particular, physicians who care for this unique population of pregnant women should be particularly experienced. Furthermore, a multi-disciplinary approach, which includes experts in the fields of Radiology, Urology, Obstetrics, and Anesthesiology, is critical to ensuring the optimal care of this complicated cohort of patients.
